# The Efficacy of a Diet Low in Fermentable Oligo-, Di-, Monosaccharides, and Polyols in Irritable Bowel Syndrome Compared to Its “Real-world” Effectiveness: Protocol for a Systematic Review

**DOI:** 10.2196/41399

**Published:** 2023-06-12

**Authors:** Sandra Jent, Natalie Sara Bez, Loan Catalano, Gerhard Rogler

**Affiliations:** 1 Department of Health Professions Bern University of Applied Sciences Bern Switzerland; 2 Department of Gastroenterology and Hepatology University of Zurich Zürich Switzerland

**Keywords:** FODMAP diet, irritable bowel syndrome, dietetics, efficacy-effectiveness gap, meta-analysis, systematic review

## Abstract

**Background:**

Irritable bowel syndrome (IBS) is associated with various gastrointestinal and nongastrointestinal symptoms and reduced quality of life. A diet low in fermentable oligo-, di-, monosaccharides, and polyols (FODMAPs) is one therapeutic option for IBS. Although the efficacy of the low FODMAP diet has been reported in several systematic reviews, the efficacy-effectiveness gap of the low FODMAP diet has not yet been assessed.

**Objective:**

This systematic review aims to compare the efficacy of the low FODMAP diet from efficacy randomized controlled trials (RCTs) with the effectiveness of studies conducted in “real-world” settings.

**Methods:**

RCTs, prospective and retrospective cohort studies, and retrospective audits assessing the low FODMAP diet in adults with IBS will be searched in 4 databases: Embase, MEDLINE, CENTRAL, and CINAHL. Two independent reviewers will perform study selection, data extraction, and risk of bias assessment and assess selected quality aspects from the Grading of Recommendations Assessment, Development, and Evaluation (GRADE) protocol. Outcomes assessed are stool frequency, stool consistency, abdominal pain, overall symptom scores, adequate symptom relief, IBS-specific quality of life, and diet adherence. Data will be summarized with forest plots without summary statistics, tables, and narrative descriptions.

**Results:**

The search, title and abstract screening, and full-text screening were completed in March 2021, and an updated search was done in May 2022. As of May 2023, data analysis is almost finished, and manuscript writing is in progress. Submission of the manuscript is expected by July 2023.

**Conclusions:**

The findings of this systematic review will compare the efficacy of the low FODMAP diet for IBS found in RCTs to the diet’s real-world effectiveness.

**Trial Registration:**

PROSPERO CRD42021278952; https://tinyurl.com/32jk43ev

**International Registered Report Identifier (IRRID):**

DERR1-10.2196/41399

## Introduction

Chronic disorders are a major contributor to the global burden of disease [1]. Many of the health problems contributing to the burden of disease involve inflammatory processes [2]. Dietary risks accounted for 255 million disability-adjusted life-years globally in 2017 [3]. Thus, nutrition and nutrition interventions play a key role in improving global health and reducing the impact of inflammatory processes [4,5].

Irritable bowel syndrome (IBS) is a chronic disease common in both Western and developing countries, affecting up to 20% of the global population [6]. It is associated with various, sometimes severe, gastrointestinal, and nongastrointestinal symptoms and often results in a reduced quality of life, reduced work performance, and increased use of health care services [7,8]. IBS belongs to the disorders of the gut-brain axis, which are characterized by abnormalities in intestinal motility, visceral sensitivity, mucosal and immune function, intestinal microbiota, and stimulus processing by the central nervous system. To this day, only symptomatic treatments are available [9].

In the past 2 decades, research has shown that a diet low in fermentable oligo-, di-, monosaccharides, and polyols (FODMAPs), or the “low FODMAP diet,” reduces gastrointestinal symptoms in patients with IBS. The rate of patients with IBS reaching a clinically relevant symptom reduction may even be higher with the low FODMAP diet than with spasmolytics [10]. Several systematic reviews have summarized available data on the efficacy of the low FODMAP diet for IBS [11-20]. The majority concluded that patients with IBS benefit from following the low FODMAP diet, in comparison to either a usual diet, a high FODMAP diet, other IBS diets, or “sham” diets [11-18]. Accordingly, several IBS guidelines now list the low FODMAP diet as one possible therapeutic intervention [21-24]. Additionally, a recent expert review on the role of diet in IBS concluded that the low FODMAP diet is “currently the most evidence-based diet intervention for IBS” [25].

The low FODMAP diet includes an initial elimination phase, during which FODMAP-rich foods are excluded to individually confirm the diet's efficacy [26]. Subsequently, the individual tolerance of the FODMAPs should be tested to allow patients with IBS to follow an individually adapted FODMAP diet [27]. The therapeutic efficacy of reducing the intake of FODMAPs is explained by decreased osmotic activity due to the remaining carbohydrates retaining less water in the intestine, reduced gas production by gut microbiota, and influences on intestinal motility [28]. Evidence on the long-term efficiency of the low FODMAP diet is still limited. Recently, studies have reported on the long-term maintenance of symptom improvement after FODMAP reintroduction. After 1 year, 65% of the participants of a small follow-up study (n=18) reported adequate symptom relief [29], and in a questionnaire study, at least 50% of participants (n=211) reported improvement of abdominal pain, bloating, wind, urgency to open bowels, and also reduced health care usage [30].

Health service research distinguishes between efficacy and effectiveness studies. The former examines the fundamental efficiency of new interventions under ideal conditions, while the latter measures the efficiency of the same interventions in everyday practice [31]. Efficacy studies tend to overestimate the efficiency because of strictly defined study populations and study procedures [32]. The magnitude of this efficacy-effectiveness gap varies from no gap detected to large differences. For example, the systematic review by Ankarfeldt et al [33] concluded that there was no efficacy-effectiveness gap on blood glucose–lowering drugs—although this may have been influenced by the limited number of included studies and different biases masking such an effect. In comparison, a meta-analysis of current systemic cancer therapies found that the median overall survival was 5.2 months less in real-world data than reported in randomized controlled trials (RCTs) [34]. To date, the potential efficacy-effectiveness gap has not been investigated in the low FODMAP diet. Therefore, the systematic review described in this protocol aims to compare the efficacy of the low FODMAP diet conducted by RCTs with the effectiveness of studies conducted in “real-world” settings.

“Real-world” refers to data collected in a setting as close as possible for usual outpatient or ambulatory treatment of patients with IBS. The term was deliberately chosen because “effectiveness studies” sometimes refer to RCTs with a broader defined population and treatment protocol but not necessarily a true representation of real-world data.

## Methods

### Overview

The review was registered with the International Prospective Register of Systematic Reviews (PROSPERO) in November 2021 (CRD42021278952). If not mentioned otherwise, this protocol has been developed based on the Cochrane Handbook for Systematic Reviews of Interventions version 6.1 [35] and the PRISMA-P (Preferred Reporting Items for Systematic Review and Meta-Analysis Protocols) reporting guidelines [36]. The guidance for the clinical evaluation of drugs for the treatment of IBS published by the United States Food and Drug Administration (FDA) [37] served as “core outcome set” but had to be adapted because of the different nature of nutrition care–related studies. Also, the FDA guideline focuses on IBS constipation and IBS diarrhea, but a range of studies investigating the low FODMAP diet included patients with IBS with all subtypes or did not report outcomes per IBS subtype.

### Eligibility Criteria

The eligibility criteria are structured according to Population, Intervention, Comparison, Outcomes, and Study Design (PICOS). To address the objective of this study, the review will apply 2 PICOS. The first includes RCTs on the low FODMAP diet's efficacy. The second focuses on the low FODMAP diet's effectiveness in real-world settings (Table 1).

**Table 1:** Study eligibility criteria based on the PICOS^a^ criteria, with time points and settings.

PICOS part		Efficacy PICOS	Real-world data PICOS
**Population**			
	Inclusion	Adults (≥18 years of age). Diagnosed with IBS^b^ according to Rome II, III and IV, NICE^c^, the German S3 guideline, Manning criteria, and Kruis score. Patients likely to represent the subjects studied in efficacy studies (eg, excluding patients with additional conditions potentially influencing the efficacy of the low FODMAP^d^ diet, such as gastrointestinal surgery). Patients with clinically relevant baseline outcome scores, allowing for relevant improvements through the intervention.	Adults (≥18 years of age). Diagnosed with IBS according to Rome II, III and IV, NICE, the German S3 guideline, Manning criteria, and Kruis score. Patients likely to represent the “usual” patients in the institution conducting the study.
	Exclusion	Animal studies. Studies on FODMAP contents and reduction of FODMAP contents in foods.	Animal studies. Studies on FODMAP contents and reduction of FODMAP contents in foods.
**Intervention**			
	Inclusion	Low FODMAP diet for at least 4 weeks. Low FODMAP diet is either carried out independently by participants or provided to them.	Low FODMAP diet for at least 4 weeks. Low FODMAP diet is carried out by participants independently in their daily life.
	Exclusion	Studies conducted with exclusive enteral nutrition. Studies investigating single FODMAPs. Studies in which the low FODMAP diet was implemented without any support from a health professional.	Studies conducted with exclusive enteral nutrition. Studies investigating single FODMAPs. Studies in which the low FODMAP diet was implemented without any support from a health professional.
**Control**			
	Inclusion	Other dietary interventions for IBS for at least 4 weeks, such as a high FODMAP diet, traditional IBS diets, sham diets, anti-inflammatory diets, probiotics, and fiber supplements. Control intervention is either carried out independently by participants or provided to them (eg, high FODMAP meals provided).	No control group is necessary. If there is a control intervention, participants need to carry it out independently at home.
	Exclusion	Studies conducted with exclusive enteral nutrition. Studies conducted with healthy controls.	Studies conducted with exclusive enteral nutrition.
**Outcomes—critical**			
	Inclusion	Stool frequency. Stool consistency. Abdominal pain.	Stool frequency. Stool consistency. Abdominal pain.
	Exclusion	N/A^e^	N/A
**Outcomes—important**			
	Inclusion	Overall symptom scores such as IBS-SSS^f^ or assessment of gastrointestinal symptoms with a visual analog scale. Number or percentage of patients with adequate symptom relief. IBS-specific quality of life. Adherence to the low FODMAP diet.	Overall symptom scores such as IBS-SSS or assessment of gastrointestinal symptoms with a visual analog scale. Number or percentage of patients with adequate symptom relief. IBS-specific quality of life. Adherence to the low FODMAP diet.
	Exclusion	N/A	N/A
**Time points**			
	Inclusion	Baseline. After the FODMAP elimination phase (sometimes also the end of intervention). After the FODMAP reintroduction (if part of the study). After the follow-up time point (if part of the study).	Baseline. After the FODMAP elimination phase (sometimes also the end of intervention). After the FODMAP reintroduction (if part of the study). After the follow-up time point (if part of the study).
	Exclusion	N/A	N/A
**Study design**			
	Inclusion	Randomized controlled trials with inclusion and exclusion criteria typical for efficacy studies (see above), including crossover trials.	Randomized controlled trials with openly formulated inclusion and exclusion criteria (see above), prospective and retrospective cohort studies as well as retrospective audits.
	Exclusion	All other types of studies.	Case reports and qualitative studies.
**Context**			
	Inclusion	Studies in any ambulatory or outpatient setting.	Studies in any ambulatory or outpatient setting.
	Exclusion	N/A	N/A

^a^PICOS: Population, Intervention, Comparison, Outcomes. and Study Design.

^b^IBS: irritable bowel syndrome.

^c^NICE: National Institute for Health and Care Excellence.

^d^FODMAP: fermentable oligo-, di-, monosaccharides, and polyols.

^e^N/A: not applicable.

^f^IBS-SSS: IBS Severity Scoring System.

The publication period is not restricted, as the first studies on the low FODMAP diet were published in 2006. Publication language is limited to English, German, French, and Italian because of practical reasons. Studies must be published as a full manuscript, or authors must provide relevant information.

The criterion of clinically relevant symptoms has been included in the efficacy PICOS based on the FDA Guidance for Industry: *Irritable Bowel Syndrome—Clinical Evaluation of Drugs for Treatment* [37]. The definition in the guidance will be applied. Alternatively, we will accept other literature-based descriptions of “clinically relevant symptoms” or when study authors have considered the baseline data as clinically relevant. Studies included in the real-world data PICOS do not need to meet this criterion, as the FDA guidance focuses on efficacy studies, and literature on instituting the low FODMAP diet in practices reports that the low FODMAP diet can be applied in mild IBS symptoms as well [26].

For the differentiation between patients in efficacy or real-world studies, we developed a rating system based on the “eligibility” domain of the Pragmatic Explanatory Continuum Indicator Summary (PRECIS-2) tool. The PRECIS-2 tool allows studies to be designed explicitly on a 5-point continuum scale (5=very pragmatic, 4=rather pragmatic, 3=equally pragmatic and explanatory, 2=rather explanatory, and 1=very explanatory) [38].

According to the literature [26], the low FODMAP diet can be applied in practice in the following situations: (1) there is a diagnosis of IBS but no “red flags” that would require further investigation; (2) mild to severe symptoms are present, and a relationship between the symptoms and the diet is assumed; and (3) there is no history of eating disorders or orthorexia.

Studies will be included in the real-world data PICOS, if they receive more than 3 points (more pragmatic), and in the efficacy PICOS, if they receive 3 points or less. This cutoff was chosen based on a pilot test of the system, in which studies scoring 3 points or less still tended to be “classic RCTs,” while those scoring more than 3 points were more pragmatic studies.

Deductions of a half or full point per aspect mentioned below will be applied if subjects are excluded because: (1) they are not expected to respond equally to the intervention (based on health conditions or dietary restrictions in the exclusion criteria list); (2) there are challenges (eg, from ethics) unrelated to the intervention (based on the exclusion of specific population groups where the low FODMAP diet would be applied in usual practice); (3) they were unable to meet strict guidelines regarding medication adjustments, including prebiotics, probiotics, and supplements; (4) they are expected to be less able to attend appointments (eg, psychological problems, lower motivation); and (5) they have not strictly adhered to the intervention.

The outcomes are divided into critical and important outcomes as recommended by the Grading of Recommendations Assessment, Development, and Evaluation (GRADE) criteria [39]; outcomes of limited importance have not been defined. The critical outcomes are based on the FDA guidance for the clinical evaluation of IBS drugs [37] but have been adapted. The important outcomes include outcomes often applied in studies on the low FODMAP diet, which are relevant from a patient’s or dietitian’s perspective. Studies will not be excluded based on the outcomes reported, but reporting will focus on the critical and important outcomes.

### Search Strategy

The search strategy includes databases: Embase (Elsevier interface, 1947 onward), MEDLINE (PubMed interface, 1966 onward), Cochrane Central Register of Controlled Trials (Wiley interface, current issue), and CINAHL (EBSCOhost interface, 1937 onward); trial registries: ClinicalTrials.gov and WHO-Portal International Clinical Trial Registry Platform; dissertations: LILACS, Open Access Theses, and Dissertations, and ProQuest Dissertation & Theses Global; gray literature; and hand searched journals: *Gut*, *American Journal of Gastroenterology*, *Journal of Gastroenterology and Hepatology*, *Gastroenterology*, *Alimentary Pharmacology and Therapeutics*, *Neurogastroenterology & Motility*, *Journal of Human Nutrition and Dietetics*, *Journal of the Academy of Nutrition and Dietetics*, *European Journal of Nutrition*, *Clinical Nutrition*, *International Journal of Clinical Practice*, *Journal of Nutrition*, and *Nutrients*.

The search will be conducted once for both PICOS categories together. The search criteria will include the terms and synonyms of FODMAPs and IBS listed below and the Medical Subject Headings (MeSH) term for IBS. At the time of the search, no MeSH term existed for FODMAPs. The search criteria will be adopted to the different databases based on the following: synonyms for FODMAPs: “FODMAP” OR “FODMAPS” OR “Fermentable, poorly absorbed, short-chain carbohydrates,” OR “Fermentable oligosaccharides, disaccharides, monosaccharides, polyols”; synonyms for IBS: “Irritable Bowel Syndromes” OR “Syndrome, Irritable Bowel” OR “Syndromes, Irritable Bowel” OR “Colon, Irritable” OR “Irritable Colon” OR “irritable bowel syndrome” (MeSH Terms).

Further restrictions in the search (eg, study design) will not be applied to not miss potentially eligible studies. Search results will be imported in Covidence (Veritas Health Innovation Ltd), a web-based software facilitating study selection, quality assessment, and data extraction in systematic reviews.

### Study Selection

**Figure 1:**
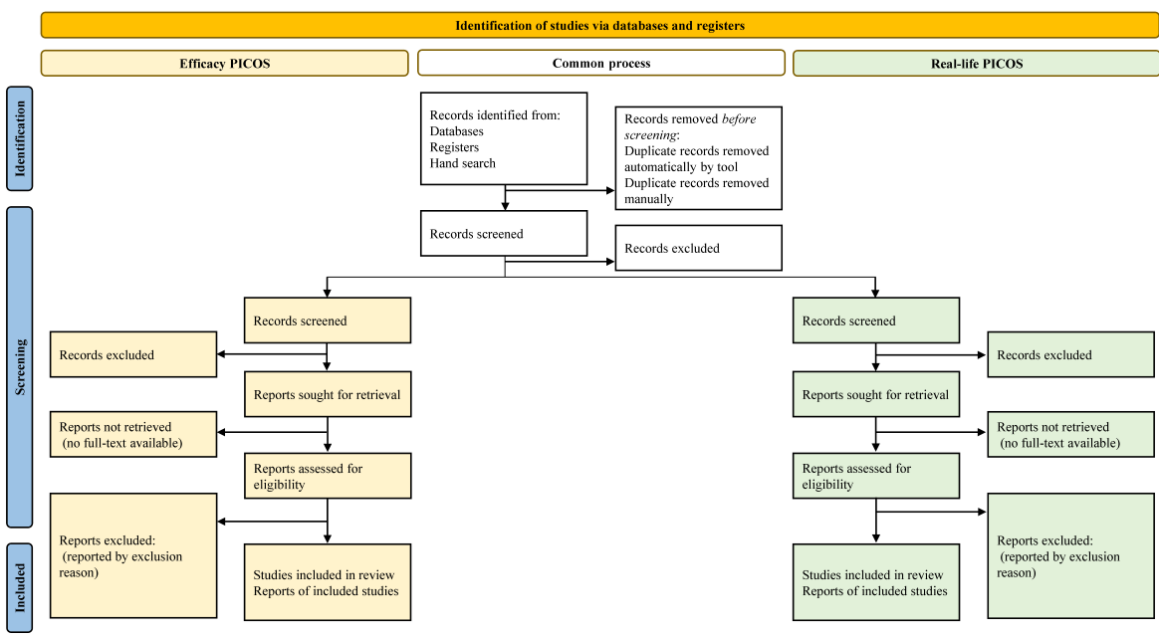
Example overview of the search strategy, reported according to the PRISMA (Preferred Reporting Items for Systematic Reviews and Meta-Analyses) statement [36]. PICOS: Population, Intervention, Comparison, Outcomes, and Study Design.

Reports found in the search process will be imported to Covidence, where duplicates detected by the system will automatically be removed. Two reviewers will do the screening independently. After each round, the 2 reviewers and the first author of this study protocol will resolve conflicts. The title and abstract screening will be done in 2 rounds. First, reports clearly not eligible for both PICOS categories will be excluded (eg narrative reviews, FODMAP content analyses, pediatric studies, and studies on other disorders than IBS). Then, the remaining reports will be separated in 2 distinct “reviews” in Covidence, and eligible studies will be determined by a second round of title and abstract screening followed by full-text screening performed for each “review” separately, based on the eligibility criteria of each PICOS (Figure 1).

### Data Extraction

Data extraction will be done in Covidence, using both predefined and added fields by the review team. Data extraction will be piloted with some studies, and educational sessions will facilitate a common understanding of data extraction. Two reviewers will independently extract the data. Conflicts will be resolved by the 2 reviewers, and the first author of this protocol will be rechecking the original information in the publications. For each study, the information found in Table 2 will be extracted. If needed, the study authors will be contacted to clarify uncertainties or be asked for additional information. Data will be transferred into RevMan (version 5.4; Cochrane Collaboration) software for data synthesis.

**Table 2:** Overview of the data to be extracted.

Covidence field	Data to be extracted
Study identification	Sponsorship, country, setting, author's contact details, year of study, aim of study, type of publication, ethical approval, and publication language.
Methods	Study design, number of centers, method of randomization, blinding, and analysis (intention to treat per protocol).
Intervention	Interventions (name for each group), intervention description, definition of symptom improvement after the intervention, duration of participation, duration of the treatment period, duration follow-up, timing, providers, and implementation.
Population	Inclusion criteria, exclusion criteria, group differences, sample size calculation, and baseline characteristics (assessed for eligibility randomized, participants per group, dropouts per group, dropout reasons, age, gender, BMI, predominant bowel habit, and diagnostic criteria).
Outcomes	Outcome measures based on the critical and important outcomes for this review, time points evaluated.

### Risk of Bias and Quality Assessment

Two reviewers will independently assess the studies’ risk of bias. In Covidence, disagreements will be resolved by rechecking the original information in the publications and by a discussion among the reviewers. Several tools will be used due to the range of studies included [35]: the revised tool for Risk of Bias in randomized trials (RoB 2) [40], the newly available test version of RoB 2 for crossover studies [41], and the Risk Of Bias In Non-randomized Studies—of Interventions (ROBINS-I) assessment tool for nonrandomized studies [42]. The RoB 2 version for crossover studies will be applied despite being a test version because it is largely identical to the original RoB 2 but has been supplemented with an additional domain for bias due to period and carryover effects [42]. We will not fully implement the GRADE system but still use specific parts of it for additional quality assessment, such as indirectness of evidence, imprecision, large magnitude of an effect, and the effect of plausible residual confounding.

### Data Analysis

Data analysis will be done in RevMan (version 5.4; Cochrane Collaboration)*.* We will not perform a meta-analysis, as we expect heterogeneous outcome measures in the real-world data PICOS. Where available, mean values of measurements of a specific period (eg, mean or median of a screening period, mean or median of the last 2 weeks of the elimination phase) will be reported. If not available, single measurements will be included. Dichotomous data (adequate symptom relief) will be reported as risk ratios and risk differences with 95% CIs. In studies without a control group, results will be presented as a percentage of participants reaching adequate symptom relief. Continuous data will be reported as “mean difference” or as “standardized mean difference” if different measures were applied, both with 95% CIs. We will also apply this to studies without a control group, using baseline data as a comparison. Results will be presented as forest plots without the summary measure, in tables, or narratively.

Data will be analyzed per PICOS separately. If at least 5 studies per subgroup can be included, we will apply the following subgroups: (1) Efficacy PICOS: diet is provided during the intervention period, elimination phase only; diet is implemented independently, elimination phase only; diet is implemented independently, elimination, and reintroduction phase; and multicomponent interventions (not only low FODMAP diet). (2) Real-live data PICOS: diet is implemented independently, elimination phase only; diet is implemented independently, elimination and reintroduction phase; and multicomponent interventions (not only FODMAP nutrition).

These subgroups have been chosen as the effects measured may be influenced by the individual factors.

### Ethical Considerations

Ethical approval is not required for this study because only data made available through publication are used for the systematic review. The results will be submitted for publication in a relevant peer-reviewed journal. Any changes from the protocol during the conduct of the systematic review will be described in the manuscript.

## Results

The literature search, title and abstract screening, and full-text screening were completed in 2021. As of May 2023, we are in the progress of finalizing data analysis and writing the publication. We expect to submit the publication by July 2023. This work has been financially supported by the “Spendenstiftung Bank Vontobel” in Zurich, Switzerland.

## Discussion

### Principal Findings

In the systematic review, we will compare the effectiveness of the low FODMAP diet in efficacy RCTs to the effectiveness of real-world data in adult patients with IBS. This comparison will focus on the following outcomes: stool frequency, stool consistency, abdominal pain, overall symptoms, adequate symptom relief, IBS-related quality of life, and adherence to the low FODMAP diet. The findings of this systematic review will allow health professionals to compare their outcomes with the results of this systematic review and potentially initiate quality improvement projects if their results are lower than expected in comparison to the results of this systematic review.

### Current Research

Research output on the effect of the low FODMAP diet is still high. Current research topics include the long-term efficiency, impact on gut microbiota, and response predictors. Data on long-term outcomes of the low FODMAP diet, however, are still scarce. Nevertheless, these data are of great importance as the effort of a restrictive 3-phase diet, such as the low FODMAP diet, is only worthwhile if symptom relief persists. Few recent studies confirmed maintenance of symptom improvement after FODMAP reintroduction with 1- or 2-year follow-up. Two recent studies reported a 50% or more symptom improvement among participants who followed an individualized low FODMAP diet after almost 1 year [29,30]. Symptom improvement may be related to continued partial adherence to the low FODMAP diet. In a retrospective cross-sectional study (n=90), almost 80% of the participants reported partial adherence after nearly 2 years, which was associated with less abdominal pain [43]. In acknowledgment of the limited RCTs available assessing long-term effect, this systematic review will still endeavor to assess all available evidence on such long-term outcomes.

There are known potential negative effects of the low FODMAP diet on the gut microbiota, due to the elimination of fructans and galactans, which are both important prebiotics. A recent meta-analysis on the effect of the low FODMAP diet on gut microbiota concluded that studies consistently reported a reduction of bifidobacteria during the low FODMAP elimination phase, but they found no indications for broad systematic changes in the gut microbiota [44]. Thus, more research on the long-term effects of the low FODMAP diet on the gut microbiota is needed. Promisingly, a small follow-up study (n=18) found no difference in bifidobacteria abundance between baseline and 1 year [29].

Evidence on predictors of symptom relief is essential as studies have reported roughly a 70% rate of satisfactory symptom relief after following the low FODMAP diet [45]. Therefore, a better understanding of such predictors would enable applying the low FODMAP diet only in patients with IBS with a probability of a higher success rate, while those with a lower chance of symptom relief could be given alternative therapies sooner. Baseline gut microbiota has been discussed as one predictor by Vervier et al [46], who identified 2 distinct microbiota clusters in patients with IBS. The health-like cluster had a gut microbiota profile similar to healthy household members, whereas the pathogenic-like cluster gut microbiota differed greatly from the healthy-household members. When eating a low FODMAP diet, gut microbiota of the pathogenic-like cluster shifted toward a more health-associated profile, and participants experienced strong symptom improvement (measured by a decrease of 194 points on the IBS Severity Scoring System). In the health-like cluster, the low FODMAP diet did not affect the gut microbiota, and participants experienced a clinically relevant but less pronounced symptom reduction (−114 points) [46]. Therefore, having a gut microbiota of the pathogenic-like cluster may lead to better symptom improvement and could be an important predictor for the benefit of the low FODMAP diet.

Systematic reviews and meta-analyses directly assessing the efficacy-effectiveness gap remain scarce. In contrast, systematic reviews seem to increasingly include observational data due to the faster availability of such data but often without sufficient differentiation between efficacy and effectiveness data [47]. Some of the systematic reviews and meta-analyses on the low FODMAP diet in patients with IBS included a range of study designs but did not clearly distinguish between these designs in data analysis [12,14-16,18]. This may be critical, especially if meta-analyses are performed [47]. This systematic review will prevent this by categorizing the included studies as either efficacy or real-world studies and summarizing the outcomes separately.

Limitations of this systematic review are related to the aim of comparing efficacy RCTs with real-world data. First, we will include RCTs in both PICOS categories, as RCTs may be clearly efficacy studies but also report on effectiveness, which would fit more in the “real-world” category. Our systematic review includes 2 PICOS to enable the differentiation of efficacy and effectiveness studies by using specific eligibility criteria representing these 2 types of studies. However, the differentiation between study populations representative of typical efficacy or effectiveness studies remains a challenge. Therefore, we developed and pretested a rating system based on the eligibility domain of the PRECIS-2 tool [38]. Based on the pretest, we are confident that the rating system will sufficiently distinguish between efficacy and real-world studies. Furthermore, the PRECIS-2 (by using all domains) has already been successfully used retrospectively by other researchers [48-50]. However, it should be acknowledged that the retrospective use of PRECIS-2 has not been well established. Second, to include as much real-world data as possible, we will include studies with different study designs in the real-world PICOS. We hypothesize that this will entail some challenges in data analysis and interpretation as some of the data will be from observational studies without control group.

### Conclusions

This protocol describes the methods of a systematic review that will compare the effectiveness of the low FODMAP diet in efficacy RCTs to the effectiveness of real-world data in adult patients with IBS. To our knowledge, this will be the first systematic review with this focus. The results will help to fill the efficacy-effectiveness evidence gap of the impact of the low FODMAP diet in adult patients with IBS and help identify research needs related to the efficacy-effectiveness gap in this field. Furthermore, the systematic review may reveal research needs with regards to long-term efficiency of the low FODMAP diet. Lastly, the systematic review will allow us to compare outcome measurements currently used in efficacy and real-world studies and whether the quality of reports on real-world studies is sufficient to compare data to efficacy studies.

## Abbreviations

FDA: United States Food and Drug Administration
FODMAP: fermentable oligo-, di-, monosaccharide, and polyol
GRADE: Grading of Recommendations Assessment, Development, and Evaluation
IBS: irritable bowel syndrome
MeSH: Medical Subject Headings
PICOS: Population, Intervention, Comparison, Outcomes, and Study Design
PRECIS-2: Pragmatic Explanatory Continuum Indicator Summary
PRISMA-P: Preferred Reporting Items for Systematic Review and Meta-Analysis Protocols
PROSPERO: International Prospective Register of Systematic Reviews
RCT: randomized controlled trial
RoB 2: Risk of Bias in randomized trials
ROBINS-I: Risk Of Bias In Non-randomized Studies—of Interventions
